# Application of mass spectrometry to elucidate the pathophysiology of *Encephalitozoon cuniculi* infection in rabbits

**DOI:** 10.1371/journal.pone.0177961

**Published:** 2017-07-19

**Authors:** Guillaume Desoubeaux, Maria del Carmen Piqueras, Ana Pantin, Sanjoy K. Bhattacharya, Roman Peschke, Anja Joachim, Carolyn Cray

**Affiliations:** 1 University of Miami - Miller School of Medicine, Division of Comparative Pathology, Department of Pathology & Laboratory Medicine, Miami, Florida, United States of America; 2 CHU de Tours, Service de Parasitologie – Mycologie – Médecine tropicale, Tours, France; 3 Université François-Rabelais, Faculté de Médecine, CEPR - INSERM U1100 / Équipe 3, Tours, France; 4 University of Miami, Mass Spectrometry Core Facility, Miller School of Medicin–, Miami, Florida, United States of America; 5 University of Veterinary Medicine, Institute of Parasitology, Department of Pathobiology, Vienna, Austria; Leibniz-Institut fur Naturstoff-Forschung und Infektionsbiologie eV Hans-Knoll-Institut, GERMANY

## Abstract

*Encephalitozoon cuniculi* is a microsporidian species which can induce subclinical to serious disease in mammals including rabbits, a definitive natural host. The pathophysiology of infection has not been comprehensively elucidated. In this exploratory study, we utilized two mass spectrometry approaches: first, the analysis of the humoral response by profiling the microsporidian antigens as revealed by Western blot screening, and second, implementing the iTRAQ^®^-labeling protocol to focus on the changes within the host proteome during infection. Seven *E*. *cuniculi* proteins were identified at one-dimensional gel regions where specific seropositive reaction was observed by Western blot, including polar tube protein 3, polar tube protein 2, and for the first time reported: heat shock related 70kDa protein, polysaccharide deacetylase domain-containing protein, zinc finger protein, spore wall and anchoring disk complex protein EnP1, and translation elongation factor 1 alpha. In addition, there was a significant increase of nine host proteins in blood samples from *E*. *cuniculi*-diseased rabbits in comparison with non-diseased control subjects undergoing various inflammatory processes. This included serum paraoxonase, alpha-1-antiproteinase F precursor and alpha-1-antiproteinase S-1 which have presumptive catalytic activity likely related to infection control, and cystatin fetuin-B-type, an enzyme regulator that has been poorly studied to date. Notably, 11 proteins were found to be statistically increased in rabbits with neurological *versus* renal clinical presentation of *E*. *cuniculi* infection. Overall, this novel analysis based on mass spectrometry has provided new insights on the inflammatory and humoral responses during *E*. *cuniculi* infection in rabbits.

## Introduction

Microsporidiosis is an opportunistic intracellular infection caused by microsporidia which are unicellular microorganisms related to fungi. It usually occurs in immunocompromised patients and is transmitted by excreta [1,2]. To date, at least 14 distinct microsporidian species have been described in humans, and some are known to be zoonotic, *e*.*g*. *Encephalitozoon cuniculi* in rabbits [3]. In this species, infection with *E*. *cuniculi* may be either subclinical or active, and resulting in various forms of encephalitozoonosis [4]. When the kidney is involved, it may lead to renal impairment, while granulomatous encephalitis is responsible for neurological signs [5]. In serious cases, especially during chronic renal failure, encephalitozoonosis can lead to fatal outcome in rabbits [6], with overall mortality rates around 50%, despite anti-*E*. *cuniculi* treatment [7]. Although the disease is very common in pet rabbits, little is known about the exact pathophysiology of *E*. *cuniculi* infection [8]. To date, most of the immunological studies have been performed in experimental models, *e*.*g*. in rats, and only rarely in the natural rabbit host [9,10]. In such a context, accurate description of the whole proteome, *i*.*e*. description of both microsporidian proteins and host proteins that are involved in the response to *E*. *cuniculi* infection, represents a novel approach to expand our understanding of this infectious agent [11].

**Fig 1:**
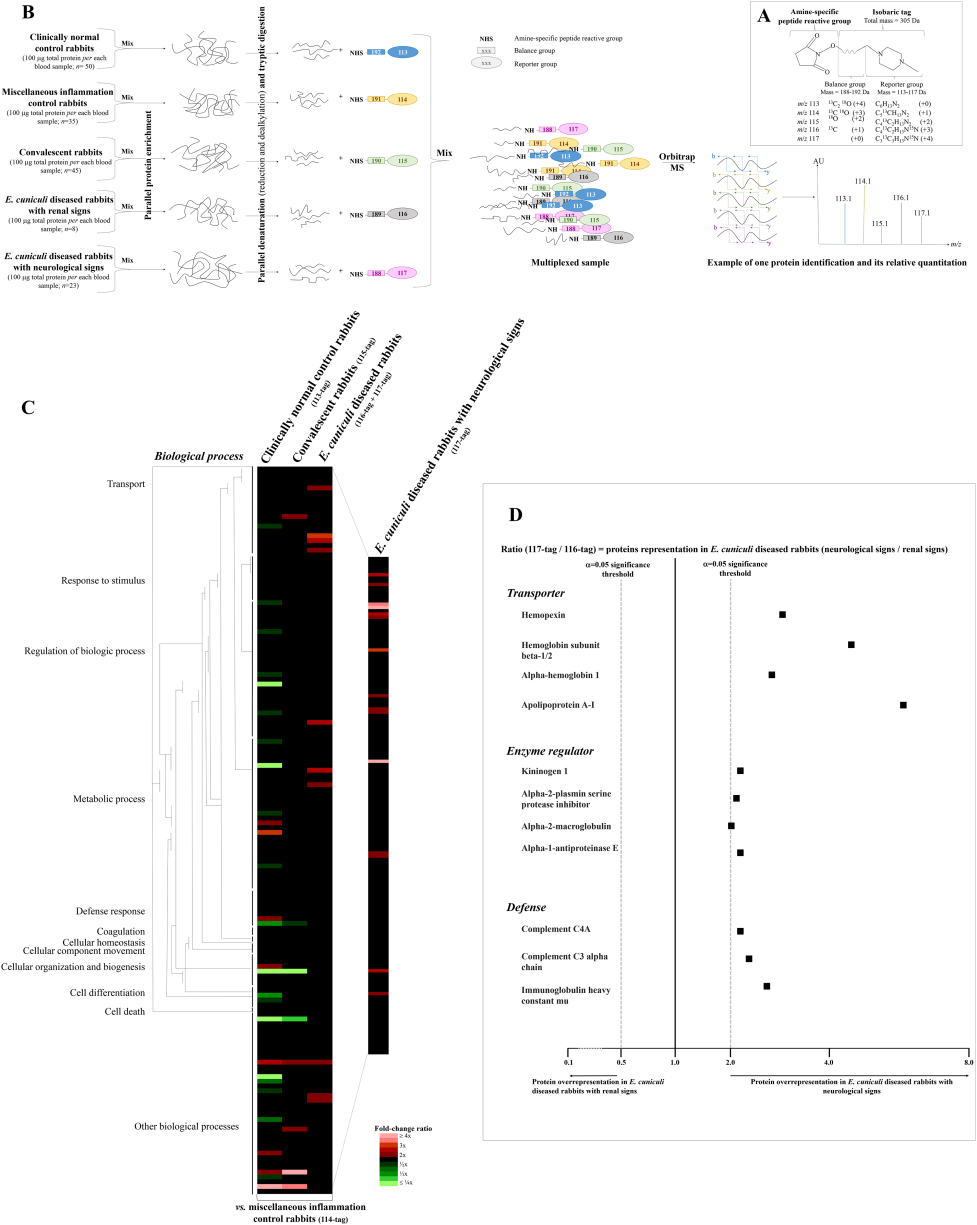
Study design and results for iTRAQ^®^ protocol. A—the iTRAQ^®^ reagent is designed as an isobaric stable tag consisting in a charged reporter group that retains charge (N,N-dimethylpiperazine), a peptide reactive group (N-hydoxy-succinimide) that is amide-linked to the N-terminus and the ε-amino side chains of all the peptides got from prior tryptic digestion, and a neutral balance portion (carbonyl) to maintain an overall mass of 305 kDa by the means of differential isotopic enrichment with ^13^C, ^15^N and ^18^O atoms. The selection of the reporter region in the low mass area enables keeping the additive mass to the fragments as negligible as possible in order to minimize any side effect during chromatographic separation and to avoid any interference with other fragment ions during mass spectrometry analysis, thus allowing for the highest degree of confidence; B—rabbit blood samples were pooled according to their clinical status. Some of them were represented in different groups (*e*.*g*. convalescent rabbits with past *E*. *cuniculi* disease but that currently underwent active inflammation due to bacteria), while other were rejected because clinical course was not clear enough. Overabundant proteins, like albumin and immunoglobulins, were removed through a commercial kit before mass spectrometry analysis. During this latter, the reporter-balance peptides remained intact, so that for one given common protein, the five samples had an identical *m/z*: the peptide fragments were equal, only the reporter ions were different. Indeed, the precursor ions and all the internal fragment ions, *i*.*e*., type b- and y-ions respectively, contain all five members of the tag set, but remain isobaric, *i*.*e*. *t*he five species have the same atomic mass but different arrangements. Thus, after collision in the mass spectrometry instrument, the five reporter group ions appeared as distinct masses ranging between *m/z* 113–117, while the remainder of the sequence-informative b- and—y ions remain isobaric and their individual current signal intensities were additive. The relative concentration of the peptides in every samples pool was then deduced from the relative signal intensities of the corresponding reporter ions; C—the hierarchical cluster diagram was constructed on the basis that, as they were considered as positive controls, non-*E*. *cuniculi* diseased rabbits with miscellaneous inflammatory processes (114-iTRAQ^®^ tag) were used as baseline. Data are visualized colorimetrically with heat plots, ‘red’ representing elevated gene expression, and ‘green’ decreased protein representation; D—the forest plot diagram shows expression levels for the 11 proteins that were found to be significantly overrepresented in *E*. *cuniculi* diseased rabbits with neurological signs (117-iTRAQ^®^ tag) in comparison with *E*. *cuniculi* diseased individuals with renal signs only (116-iTRAQ^®^ tag). Abbreviations: b-ion, precursor ion; C, Carbon; Da, Dalton; MS, Mass spectrometry; *m/z*, mass-to-charge ratio; O, Oxygen; N, azote; y-ion, internal ion.

Mass spectrometry (MS) platforms with modern devices and analytical capacity can rapidly address proteomics in any fluids or tissues. Highly-performance instruments, like Quantitative Ion Trap—Time Of Flight (QIT-TOF) or Orbitrap technologies, have recently been developed [12], and innovative ready-to-use commercial reagent kits have progressively replaced the conventional electrophoresis gels for pre-processing steps [13]. For example, isobaric Tagging for Relative and Absolute Quantitation (iTRAQ^®^) protocol is a new labeling method used in quantitative proteomics to identify and to determine, during a single-run experiment, the relative changes of individual proteins representation within different biological specimens [14–16]. The iTRAQ^®^ protocol uses distinct stable isotope-labeled molecules of varying mass that can be covalently bounded to the N-terminus and side chain amines of all the peptides obtained after protein digestion of one sample (Fig 1A). Once labelled, all the processed specimens are thereafter pooled, and the tagged peptides of this multiplexed solution are thereafter fractionated by liquid chromatography, and analyzed by MS tandem (MS/MS). The peak intensities of every obtained ion are then compared in order to calculate the relative abundance of each peptide, and, consequently, of the protein from which they are derived.

In the current study, we utilized two MS tools. First, protein profiling evaluated the primary antigens targeted by the humoral immune response as revealed by Western blot (WB), and second, the iTRAQ^®^ protocol was used on different groups of infected or non-infected rabbits to study the proteomic changes that occur in the blood of animals naturally infected with *E*. *cuniculi*. This approach allowed identification of seven antigenic microsporidian proteins and nine host proteins with increased expression during encephalitozoonosis.

## Material and methods

### Study population

Rabbit blood samples were obtained from a national multicenter study which was described in detail in previous report [17]. It systematically included patients with suspected encephalitozoonosis at time of their first visit in one of 46 veterinary clinics in the United States (U.S.) over two periods, Feb 2015 –Aug 2015 and Feb 2016 –Aug 2016. Adjustment on control subjects was made by matching on age ± 1 year, sex, and total protein concentration ± 0.5 g/dL, initially in a ratio 1: 1.

### Identification of microsporidian proteins involved in the humoral immune response in rabbits with presumptive *E*. *cuniculi* infection

#### Preparation of *E*. *cuniculi* protein extraction

The *E*. *cuniculi* proteins were extracted from strain CH-K 2169 cultured in Madin-Darby Canine Kidney (MDCK) cells (ATCC^®^ Number CCL-34), as described previously [17,18]. After a washing step in Hank´s Balanced Salt Solution (HBSS, PAA, Pasching, Austria), followed by a treatment with 0.25% sodium dodecyl sulfate (SDS) to dissolve the MDCK cells and three washing steps with HBSS, *E*. *cuniculi* spores were disrupted in a reducing buffer containing 0.0625 M Tris-HCl and 2% SDS by homogenization, followed by thawing-freezing cycles in liquid nitrogen, then centrifugation. The extracted protein fraction was stored in 100 mM dithiothreitol (DTT) and 2.5% sodium dodecyl sulfate (SDS) suspension for 72 h at 4°C, and -20°C until usage.

#### SDS-PAGE with extracted *E*. *cuniculi* proteins and Western blotting

After heating for 5 min at 95°C, the abovementioned protein fraction was separated on a 12% SDS-polyacrylamide gel electrophoresis (SDS-PAGE) in SDS-reducing buffer containing 5% ß-mercapto-ethanol, following the running conditions: 200 V, 200 mA, 6 W, during 1 h 30 min, as described previously [17,18]. For WB preparation, blood samples, 1: 300 diluted in Tris-buffered saline and Polysorbate 20 buffer, were incubated with strips cut from pure nitrocellulose membrane (pore size 0.45 μm; Bio-Rad, Hercules CA, USA) on which the separated *E*. *cuniculi* protein fraction was previously electrophoretically transferred at 35 V, 5 W, 150 mA, for 3 h [17,18]. Immunodetection was finally completed as previously reported [17]. Samples were performed in triplicate and independently read by two experts. For every run, a highly *E*. *cuniculi*-positive control serum was used as reference.

#### Processing step for the *E*. *cuniculi* proteins by mass spectrometry profiling

WB bands that were shown to be involved in the immune response in the previous step (sensitivity and/or specificity ≥ 80.0% [17]) were compared to SDS-PAGE gel stained with Coomassie-blue. Briefly, pieces of gel were individually cut into 1 mm^3^ rectangles at positions corresponding to the molecular weight (MW) of interest, as shown by WB interpretation. After 30 min washing in 1: 1 acetonitrile and ammonium bicarbonate solution, 10 mM DTT/100 mM ammonium bicarbonate were added to cover the gel pieces during 45 min at room temperature, and then replaced by 55 mM iodoacetamide (GE Healthcare, Little Chalfont, UK)/100 mM ammonium bicarbonate in the dark for 30 min. Washing steps and drying in an Eppendorf Vacufuge^®^ concentrator (ThermoFisher Scientific, Waltham MA, USA) were followed by trypsin digestion with 0.1 μg/μL sequencing-grade modified trypsin (Promega, Madison WI, USA) in 15 mM N-ethylmorpholin (Sigma-Aldrich, Saint-Louis MO, USA) for 4 h at 37°C. Peptides were thereafter extracted by 10 min incubation in acetonitrile and dried in Eppendorf Vacufuge^®^ concentrator. Digestion mixtures were re-suspended in a 2% dilution of acetonitrile and water that are suitable for ultra-high performance liquid chromatographic method (UHPLC). UHPLC was conducted according to the manufacturer’s indications in an Easy Nano LC 1000^®^ model (ThermoFisher Scientific, Waltham MA, USA) where a 75 μm i.d. x 15 cm column, packed with Acclaim PepMap^®^ RSLC C18-2 μm 100 Å column (ThermoFisher Scientific, Waltham MA, USA), was in-line connected to a Acclaim PepMap^®^ 100 75 um x 2 cm, nanoviper C18-3 μm 100 Å pre-column (ThermoFisher Scientific, Waltham MA, USA). Peptides were eluted following a 60 min gradient from 2% to 98% acetonitrile (ThermoFisher Scientific, Waltham MA, USA) in UHPLC-UV water at a flow of 350 nL/min. Then, peptides were run in a QExactive^®^ Orbitrap mass spectrometer (ThermoFisher Scientific, Pittsburg PA, USA) in a data-dependent mode, with an automatic gain control (AGC) target of 1.0x10^6^ for full MS at 70,000 resolution, and of 5.0x10^5^ for dd-MS^2^ at 17,500 resolution in positive mode. The isolation window was fixed to 1.5 *m*/*z* with normalized collision energy (NCE) of 28 eV, underfill ratio of 1.0%, and dynamic exclusion of 3.0.

#### Mass spectrometry data analysis and identification of *E*. *cuniculi* proteins

The bioinformatics analysis was performed using the Proteome Discoverer^®^ software (ThermoFisher Scientific, Pittsburg PA, USA). Specific SwissProt^®^ reviewed non-redundant databases (http://www.uniprot.org/uniprot/) were used for the analysis of *E*. *cuniculi* proteome using the Sequest^®^ HT search engine (Washington DC, USA). The parameters of the study set trypsin as the enzyme used for digestion, with a maximum number of missed cleavage sites of two, the minimum peptide length was set to six, and the maximum to 144. Maximum value for delta Correlation (Cn) was set to 0.05. False discovery rate (FDR) target value was 0.01 for strict rate and 0.05 for relaxed one, considering the *q*-value as a validation reference. Each protein identification was checked by comparing the MW proposed by Sequest^®^ with the observed value obtained on a Coomassie-blue stained SDS-PAGE.

### Analysis of the expression changes of host proteins in rabbits with presumptive *E*. *cuniculi* infection

#### Group definition and pooling

For each experimental group, blood samples were pooled into a single tube by including 100 μg protein for each rabbit, in order to constitute a representative sample. The groups were defined as follows: clinically normal control rabbits, control rabbits undergoing miscellaneous inflammatory conditions, rabbits with a medical history of infection compatible with convalescence and/or recovery following specific anti-*E*. *cuniculi* treatment, and diseased rabbits with presumptive active *E*. *cuniculi* infection. This latter group was subdivided in two pools based on clinical presentation: the first including rabbits showing only renal signs and the second including rabbits with neurological signs (Fig 1B). Rabbits with complex clinical courses, including multiple overlapping signs, and those with ocular disease, were excluded.

#### Pre-processing step for host proteins with iTRAQ^®^ labeling

Protein enrichment was completed in each of the five tubes using the Pierce Albumin / IgG removal^®^ kit (ThermoFisher Scientific, Waltham MA, USA) that allows for depletion of overrepresented proteins like albumin or immunoglobulins [8,16]. Protein was quantified in each pool in a Pierce BCA Protein assay^®^ kit (ThermoFisher Scientific, Waltham MA, USA), according to the manufacturer’s instructions [16,19]. Then, 100 μg of each sample pool was incubated with 30 μL dissolution buffer of 0.5 M triethylammonium bicarbonate (Sigma-Aldrich, Saint-Louis MO, USA) at pH 8.5, and thereafter treated with 2% SDS-denaturant solution. One microliter of tris-(2-carboxyethyl) phosphine-reducing reagent was added, followed by vortex-shaking for 1 min, centrifugation at 18.000 x *g* for 5 min and incubation at 60°C for 1 h. After another step of shaking and centrifugation, 84 mM iodoacetamide was added for a 30 min incubation in the dark at room temperature. Freshly prepared 0.1 μg/μL sequencing grade modified trypsin was them mixed with each sample, and digestion was carried out at 37°C during 30 min. Thereafter, 1 μL trypsin was added to continue digestion overnight. The samples were dried in an Eppendorf Vacufuge^®^ concentrator, then reconstituted with 30 μL dissolution buffer. Every tube was mixed with one iTRAQ^®^ reagent vial (Sigma-Aldrich, Saint-Louis MO, USA) and reconstituted in isopropanol, according to the manufacturer’s recommendations. The tube containing sample aliquots from healthy subjects was mixed with the 113-reagent, those from controls with inflammation due to other reasons with the 114-reagent, those from convalescent infected rabbits with the 115-reagent, those from animals with encephalitozoonosis exhibiting renal signs with the 116-tag, and those with neurological signs with the 117-reagent (Fig 1B). Finally, the contents of all the iTRAQ^®^ reagents-labelled tubes were combined into one single tube, vortexed for 1 min, and centrifuged at 18,000 *g* for 5 min. The multiplexed specimen was totally dried by centrifugal vacuum concentration as above.

#### Processing steps for the host proteins by mass spectrometry iTRAQ^®^ protocol

The aliquot of the mixed iTRAQ^®^ sample was re-suspended in 2% acetonitrile and loaded onto the UHPLC instrument as described above. Peptides were eluted following a 75 min-gradient from 2% to 98% acetonitrile. After elution, the resolved peptides were collected and run in the QExactive^®^ Orbitrap mass spectrometer, as described above, with an AGC target of 1.0x10^6^ for full MS, and 2.0x10^5^ for dd-MS^2^. The isolation window and the NCE were the same as above. First mass was fixed to 103 *m/z*. Each aliquot was tested in triplicate.

#### Mass spectrometry data analysis and identification/quantitation of host proteins

Same Proteome Discoverer^®^ software and SwissProt^®^ databases were used as described above, allowing only two missed cleavages, 10 ppm as precursor mass tolerance, and 0.02 Da for fragment mass tolerance. Alkylation, as well as N-terminal / lysine modifications and dynamic iTRAQ^®^ modifications were selected as fixed, and ion score or expected cut-off < 0.05 (95% confidence). Strict and relaxed FDR were set to the same values as reported above. At the quantification level, the analysis included Κ-means clustering, *i*.*e*. supervised and heuristic algorithm, for both protein and peptide quantitation ratios. The software actually worked by grouping proteins, based on the peptide spectral match (PSMs). Then, a customized ratio was calculated for every protein group as the median of all PSMs included in the protein group. Only ratios with *P* < 0.05 and only fold-changes ≥ 2.0 across two aliquot replicates were used to determine up- or down-regulated proteins [20].

### Statistical analysis

Statistical analyses were performed using XLStat^®^ v.2016.6.04 software (Addinsoft, Paris, France). Missing data, *e*.*g*. when the total volume of sample was insufficient to complete all the analyses, were managed by the method of mean imputation. The α-risk was adjusted at 0.05.

Regarding the iTRAQ data, we based inference on one-way analysis of variance (ANOVA) models, with the data analysis conducted one protein at a time [21,22], combining both normalization, *i*.*e*. bias removal, and assessment of differential protein expression in a single model fit to the collection of reporter ion peak areas, *i*.*e*. corrected for isotopic overlap, from all observed tandem mass spectra. Principal component analysis (PCA) was used to facilitate interpretation of the multivariate proteome dynamics dataset [23].

### Ethics

No animal was anesthetized, euthanized, or sacrificed upon purposes of this study. The serum samples used herein were remainder parts of the routine vet work, and thus do not require specific ethical approval. However, this study was carried out in strict accordance with the recommendations in the *Guide for the Care and Use of Laboratory Animals of the National Institutes of Health* and with the *Institutional Animal Care & Use Committee* (IACUC) of University of Miami-Research. All the local ethic committees of submitting vet facilities agreed with the protocol of this study.

## Results

### Study population

Overall, samples from 153 rabbits were enrolled. This included 72 rabbits with the presumptive diagnosis of encephalitozoonosis. Of those subjects which were treated with *E*. *cuniculi*-directed therapies, 86.8% recovered. Eighty-one patients were included as control population, with 67.9% showing various signs related to miscellaneous infections or inflammatory processes but not related to active encephalitozoonosis, *e*.*g*. bacterial kidney infection, otitis, arthritis, spine injury, although 55.5% of them also had anti-*E*. *cuniculi* antibodies due to past infection. The remaining 26 rabbits in the control group were seronegative and clinically normal. The demographics of the patients are summarized in Table 1.

**Table 1:** Overall characteristics of the included rabbit patients in the final cohort. The group assignment was made according to the method reported in the *Material & Methods section*.

	Mean (± standard deviation) or *N* (%), [_95%_CI]	
Study population (*N* = 153)	*E*. *cuniculi* diseased cases (*N* = 72)	Non-*E*. *cuniculi* controls^ψ^ (*N* = 81)
**Age** (years)	**5.85** y (±2.9), [5.2–6.5 y]	**4.3** y (±3.7), [3.5–5.2 y]
**Gender** (male sex)	**43** (59.7%), [48.4–71.1%]	**46** (56.8%), [46.0–67.6%]
**Clinical course** including the following symptoms^″^	**61** (84.7%) [76.4–93.0%]	**55** (67.9%), [57.7–78.1%]
- neurological^1^	36 (55.4%), [43.3–67.5%]	35 (55.6%), [43.3–67.8%]
- digestive^2^	16 (24.6%), [14.1–35.1%]	15 (23.8%), [13.3–34.3%]
- renal^3^	14 (21.5%), [11.5–31.5%]	3 (4.8%), [0.0–10.0%]
- ocular^4^	11 (16.9%), [11.5–31.5%]	5 (7.9%), [1.3–14.6%]
- other^5^	9 (13.8%), [5.5–22.2%]	6 (9.5%), [2.2–16.8%]
**Biological findings:**		
- IgG ELISA titer (1:dilution)	**1024** (1792), [0–8192]^¥^	**0** (0), [0–2048]^¥^
- IgM ELISA titer (1:dilution)	**128** (96), [0–512] ^¥^	**0** (0), [0–256]^¥^
- total protein concentration (g/dL)	**7.4** (±3.1), [6.7–8.1]	**7.8** (±3.1), [7.1–8.5]
C-reactive protein (ng/mL)	**39.3** (±54.2), [26.6–52.0]	**7.1** (±29.2), [0.6–13.5]
**Anti-*E*. *cuniculi* curative therapy** including the following drug(s)^⟡^:	**53** (73.6%) [63.4–83.8%]	
- oxibendazole	9 (14.5%), [5.7–23.2%]	
- fenbendazole	29 (46.8%), [34.4–59.2%]	
- albendazole	2 (3.2%), [0.0–7.6%]	
- other	13 (21.0%), [10.8–31.1%]	
**Total duration of anti-*E*. *cuniculi* treatment** (days)	**83.1** d (±137.9), [43.1–123.2 d]	
**Clinical outcome** (survival)	57* (79.2%), [69.8–88.5%]	**76**^#^ (93.8%), [88.6–99.1%]

Abbreviations: _95%_CI, 95% confidence interval; ELISA, Enzyme-Linked Immunosorbent Assay; *N*, number; deviation; /, 0 (0.0%), [0.0–0.0%].

^**ψ**^ included clinically normal control rabbits and control rabbits undergoing miscellaneous inflammatory conditions

^**⟡**^ associations are possible

^1^ *i*.*e*., head tilt, ataxia, circling, nystagmus, rotational / rolling / swaying movements, torticollis, seizure, cortical blindness, abnormal spine reflexes, paresis, head tremors / nodding

^2^ *i*.*e*., chronic/recurring gastrointestinal disease (*e*.*g*. stasis), weight loss

^3^ *i*.*e*., polyuria, polydipsia, dehydration, osteodystrophy

^4^ *i*.*e*., unilateral phacoclastic uveitis, cataracts, endophthalmitis

^5^ *i*.*e*., lethargy, aggression, auto-mutilation, excessive running / jumping

^**¥**^ median (interquartile range), [min.–max. value]

* 86.8% which received specific anti-*E*. *cuniculi* therapy improved their health status; four patients were lost to follow up

^#^ two patients were lost to follow up

### Identification of microsporidian proteins involved in the humoral immune response in rabbits with presumptive *E*. *cuniculi* infection

**Fig 2:**
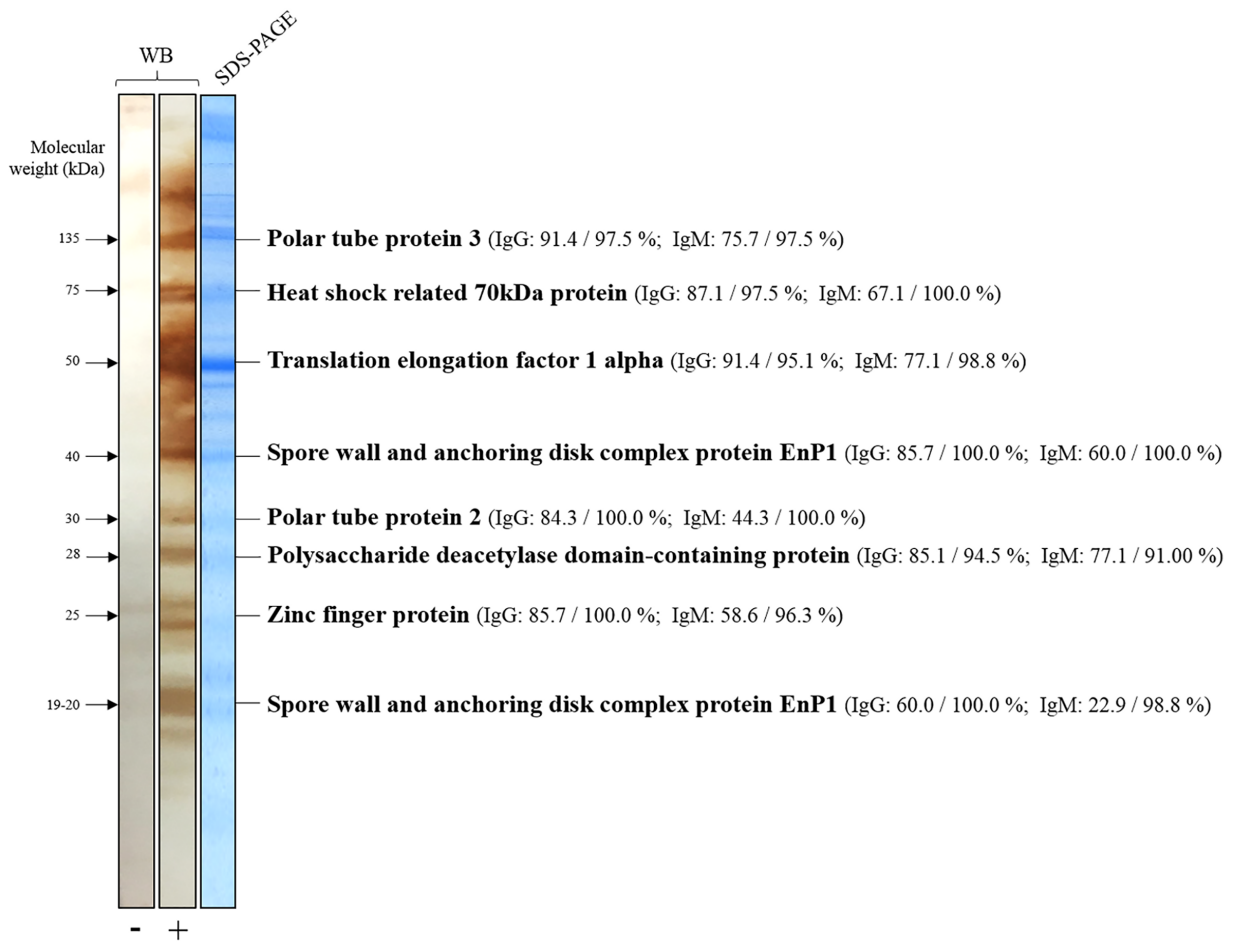
Example of negative and positive Western blot (WB) patterns for extracted *Encephalitozoon cuniculi* proteins using goat anti-rabbit IgG conjugate. The identification of the proteins in the SDS-PAGE gel (right panel) corresponding to the bands of interest on the WB (left panel) are listed on the right. In brackets: the respective sensitivity and specificity for IgG and IgM detection for each WB band. The protein identified as spore wall and anchoring disk complex protein EnP1 at 17–19 kDa was actually a fragment of the one found in 40 kDa. Abbreviations: -, Negative; +, Positive; kDa, Kilodalton; WB, Western blot.

Eight WB bands were retained for further MS analysis, as they were consistently observed in samples tested from seropositive animals with clinical signs and biological evidence of *E*. *cuniculi* infection. They were located at approximately 135, 75, 50, 40, 30, 28, 25 and 19–20 kDa (Fig 2). Noteworthy by WB, the following antibodies were found to be highly-specifically associated with active encephalitozoonosis expressing clinical signs rather than a past infection: IgM antibodies directed against the protein located at 170kDa (odds ratio (OR) = 4.0, 95% confidence interval (_95%_CI)[1.3–12.3], *P* = 0.02), against the protein at 75kDa (OR = 8.6, _95%_CI [2.7–27.4], *P* = 0.0003), against the 50kDa protein (OR = 8.2, _95%_CI [2.4–27.7], P = 0.0008), against the 40kDa protein (OR = 5.8, _95%_CI [2.0–16.9], *P* = 0.001), against the 28kDa protein (OR = 4.1, _95%_CI [1.4–12.9], *P* = 0.02), and the 25kDa protein (OR = 5.5, _95%_CI [1.9–15.9], *P* = 0.002). During active infection, IgG antibodies were directed against the protein located at 50kDa on Western blotting (OR = 27.9, _95%_CI [4.2–187.9], *P* = 0.0006) and the protein at 30kDa (OR = 5.0, _95%_CI [1.3–19.5], *P* = 0.02). However, no differences in WB patterns were observed between rabbits exhibiting renal or neurological signs.

MS successfully identified seven proteins in regions of the Coomassie-blue gel that corresponded to the bands detected in Western blotting (Table 2): heat shock related 70 kDa protein, translation elongation factor 1 alpha, zinc finger protein, polysaccharide deacetylase domain-containing protein, polar tube protein 3, polar tube protein 2, and spore wall and anchoring disk complex protein EnP1, a fragment of which was also found at 19–20 kDa.

**Table 2:** List of the microsporidian proteins which were located in regions of the Coomassie-blue SDS-PAGE gel that corresponded to the bands detected in Western blotting analysis using sera from the rabbits infected with *Encephalitozoon cuniculi*.

No. WB band	Protein identification	Known function	Chromosome	Gene name (GenBank^®^)	Access. Number (UniProt^®^)	Subcellular location	Theoretical MW (kDa)	Calc. pI	Protein score (Sequest^®^)	No. unique peptide(s)	No.aa	% Seq.coverage
**135**	**Polar tube protein 3**	**cell differentiation** (sporulation resulting in formation of a cellular spore)	XI	ECU11_1440	Q8MTP3	spore polar tube	136.1	6.2	3,351	67	1,257	70
**75**	**Heat shock related 70kDa protein**	**metabolic process** (stress response); cell **organization and biogenesis** (nucleotide binding)	III	ECU03_0520	Q8SSB1	cytoplasm	74.8	5.5	854	60	683	39
**50**	**Translation elongation factor 1 alpha**	**metabolic process** (catalytic activity); **cell organization and biogenesis** (nucleotide binding; RNA binding)	IV	ECU04_1100	Q8SS29	cytoplasm	51.6	8.8	495	23	471	51
**40**	**Spore wall and anchoring disk complex protein EnP1**	**cell differentiation** (cell adhesion and sporulation resulting in formation of a cellular spore)	I	ECU01_0820	Q8SWL3	spore wall	40.6	9.1	412	12	357	28
**30**	**Polar tube protein 2**	**cell differentiation** (sporulation resulting in formation of a cellular spore)	VI	ECU06_0240	Q8SRT0	spore polar tube	30.1	8.4	199	12	277	27
**28**	**Polysaccharide deacetylase domain-containing protein**	**metabolic process** (catalytic activity)	XI	ECU11_0510	Q8SU65	Intramembranous space	28.1	4.7	159	7	254	33
**25**	**Zinc finger protein**	**transport** (metal-ion binding)	IV	ECU04_1480	Q8SVQ9		23.8	5.6	79	5	214	17
**19–20**	**Spore wall and anchoring disk complex protein EnP1**	**cell differentiation** (cell adhesion and sporulation resulting in formation of a cellular spore)	I	ECU01_0820	Q8SWL3	spore wall	40.6	9.1	60	5	357	17

Abbreviations: aa, Amino acids; Access., Accession; calc., Calculated; kDa, Kilodalton; MW, Molecular weight; No, Number; pI, Isoelectric point; Seq., Sequence; WB, Western blot.

### Analysis of the changes of host proteins in rabbits with presumptive *E*. *cuniculi* infection

Detailed composition of every iTRAQ^®^ group is summarized in Fig 1B. According to their clinical status, global expression patterns for host proteins were quite distinct between the different groups of patients and the controls (Fig 1C). Nine proteins were found to be significantly increased in diseased rabbits with presumptive *E*. *cuniculi* infection (Table 3). Five of these proteins have a putative catalytic activity: alpha-1-antiproteinase F precursor, alpha-1-antiproteinase S-1, serum paraoxonase/arylesterase, haptoglobin and ceruloplasmin, while the two latter are also theoretically involved in protein and metal-ion binding, respectively. Cystatin fetuin-B-type is an enzyme regulator which was 2.6-fold overexpressed in diseased subjects. Two other proteins with increased expression have transporter activity and are able to bind iron ion: hemoglobin subunit beta-1/2 and alpha-hemoglobin were increased 3.1- and 2.4-fold, respectively.

**Table 3:** List of the host proteins which were shown to have significantly increased expression (≥ 2-fold) during disease in rabbits infected with *Encephalitozoon cuniculi*.

Protein identification	Foldchange	Known function	Chromosome	Gene name (GenBank^®^)	Access.number (UniProt^®^)	Subcellularlocation	Theoretical MW (kDa)	Calc.pI	Proteinscore (Sequest^®^)	No.uniquepeptide(s)	No.aa	% Seq.coverage
**Alpha-1-antiproteinase F precursor**	2.1	**metabolic process** (catalytic activity)		*LOC100008973*	G1TFV7	extracellular space	45.8	6.2	508	2	413	48
**Alpha-1-antiproteinase S-1**	2.4	**metabolic process** (catalytic activity)		***LOC100008980***	Q07298	extracellular space	45.7	6.4	521	1	413	46
**Ig gamma-chain C region**	2.5	**defense response and transport** (protein binding)		*LOC100009097*	G1THZ6	extracellular space	51.9	7.7	104	2	480	12.6
**Haptoglobin**	2.4	**metabolic process (catalytic activity); transport (protein binding); regulation of biological process; response to stimulus**	V	***HP***	G1SWF6	extracellular region	38.9	6.3	171	12	347	29.7
**Serum paraoxonase/ arylesterase**	2.6	**metabolic process (catalytic activity); transport (metal-ion binding, protein binding); response to stimulus; regulation of biological process**	X	***PON1***	G1SN96	blood microparticle; extracellular; spherical high-density lipoprotein particle	38.2	5.6	113	1	342	38
**Cystatin fetuin-B-type**	2.6	**regulation of biological process** (enzyme regulator activity)	XIV	***FETUB***	G1SEK8	extracellular exosome; extracellular space	42.9	5.9	146	7	392	15
**Ceruloplasmin**	2.0	**metabolic process (catalytic activity); transport (metal-ion binding); cellular homeostasis**	XIV	***CP***	G1SJX3	cell	121.1	5.6	164	20	1067	24
**Hemoglobin subunit beta-1/2**	3.1	**transport** (transporter activity, metal ion binding)	I	***HBB1 / HBB2***	P02057 / A0A140TAV6	hemoglobin complex	16.1	8.2	456	4	147	79
**Alpha-hemoglobin**	2.4	**transport** (transporter activity, metal ion binding)		***HBA***	B8K132	hemoglobin complex	15.6	8.2	208	1	142	51

Fold changes in the 31 diseased rabbits (*i*.*e*. encompassing all the subjects for which blood samples were tagged with either 116- or 117-labels) were calculated with respect to the protein expression in the 35 non-diseased rabbits undergoing non-*E*. *cuniculi* inflammation due to any miscellaneous cause (114-tag reagent).

Abbreviations: aa, Amino acids; Access., Accession; calc., Calculated; kDa, Kilo dalton; MW, Molecular weight; No, Number; pI, Isoelectric point; Seq., Sequence

Eleven proteins were found to have increased expression in rabbits with neurological clinical signs in comparison with kidney encephalitozoonosis (Fig 1C and 1D; Table 4). This included hemoglobin subunit beta-1/2 and alpha-hemoglobin. Two additional transporter proteins, hemopexin and apolipoprotein A-I, were also found to be increased as well as three proteins that are presumably involved in the immune response: complement C4A, complement C3 alpha chain and immunoglobulin heavy constant mu, according to 2.2-, 2.3- and 2.7-fold changes respectively. Four enzyme regulators were increased concomitantly with presence of neurological signs: kininogen I, alpha-2-plasmin serine protease inhibitor, alpha-2-macroglobulin and alpha-1-antiproteinase E.

**Table 4:** List of the host proteins which were shown to have significantly increased expression (≥ 2-fold) in rabbits with neurological *E*. *cuniculi* disease. Fold changes of the 117-tagged proteins in the 23 rabbits with neurological signs due to *E*. *cuniculi* infection were calculated with respect to the protein expression in the eight rabbits with renal signs (116-tag).

Protein identification	Fold change	Known function	Chromosome	Gene name (GenBank^®^)	Access. number (UniProt^®^)	Subcellular location	Theoretical MW (kDa)	Calc. pI	Protein score (Sequest^®^)	No. unique peptide(s)	No. aa	% Seq. coverage
**Hemopexin**	3.1	**transport** (transporter activity, metal-ion binding); **cellular homeostasis; regulation of biological process; metabolic process**	I	***HPX***	P20058/ G1TVS4	cell; extracellular space	51.7	7.2	255	1	460	29
**Hemoglobin subunit beta-1/2**	4.4	**transport** (transporter activity, metal-ion binding)	I	*HBB1 / HBB2*	P02057/ A0A140TAV6	hemoglobin complex	16.1	8.2	456	4	147	79
**Alpha-hemoglobin 1**	2.9	**transport** (transporter activity, metal-ion binding)		*HBA1*	B8K132 /G8ZF18	hemoglobin complex	15.6	8.2	207	1	142	51
**Kininogen 1**	2.2	**regulation of biological process** (enzyme regulator activity); **transport** (protein binding); **cellular homeostasis**	XIV	***KNG1***	G1SN00	blood microparticle; cell; extracellular exosome	48.6	6.3	91	8	439	22
**Alpha-2-plasmin serine protease inhibitor (serpin family F member 2)**	2.1	**transport** (protein binding); **regulation of biological process** (enzyme regulator activity); **cell organization and biogenesis**	XIX	***SERPINF2***	Q45GR2	blood microparticle; cell surface; extracellular exosome; fibrinogen complex	54.7	6.3	198	12	491	28
Alpha-2-macroglobulin*	2.0	**regulation of biological process** (enzyme regulator activity)	VIII	*A2MG*	G1SQV9	extracellular space	165.3	7.1	624	36	1490	27
**Apolipoprotein A-I**	6.3	**transport** (transporter activity, protein binding); **regulation of biological process** (enzyme regulator activity); **metabolic process**	I	***APOA1***	P09809	extracellular space; high density lipoprotein particle	30.6	5.7	599	2	266	70
**Complement C4A**	2.2	**regulation of biological process** (enzyme regulator activity); **metabolic process; defense response and transport** (protein binding); **response to stimulus**	XII	***LOC100356976***	G1SS91	extracellular space	192.7	7.1	148	15	1752	13
**Complement C3 alpha chain**	2.3	**regulation of biological process** (enzyme regulator activity); **metabolic process; defense response and transport** (protein binding); **response to stimulus**		*C3*	P12247	extracellular space	81.8	5.7	124	1	726	25
**Immunoglobulin heavy constant mu**	2.7	**defense and transport** (protein binding)		*LOC100339957*	G1T5L0	membrane	49.9	5.9	284	9	454	26
**Alpha-1-antiproteinase E**	2.2	**transport** (protein binding); **regulation of biological process** (enzyme regulator activity)		***LOC100008986***	Q28665	extracellular space	45.7	6.1	491	1	413	46

Abbreviations: aa, Amino acids; Access., Accession; calc., Calculated;

*, homologous protein; kDa, Kilo dalton; MW, Molecular weight; No, Number; pI, Isoelectric point; Seq., Sequence.

In convalescent individuals with past medical history of *E*. *cuniculi* infection, four proteins were significantly upregulated and not observed at these levels in rabbits with current active infection (Fig 1C, Table 5): one intra-Golgi vesicle membrane uncharacterized protein, Ig gamma chain C-region, immunoglobulin heavy-chain variable region and nudix hydrolase 11. Furthermore, in the same convalescent subjects, three proteins were all found to be largely decreased in comparison to control rabbits with *E*. *cuniculi*-unrelated inflammation and to diseased rabbits with active infection: SET domain bifurcated 2 protein, which is an histone-lysine N-methyltransferase, DEAH (Asp-Glu-Ala-His) box polypeptide 29, which is an ATP-dependent helicase of the 43S ribosomal preinitiation complex in the mitochondria, and keratin 1, which is a structural protein also involved in negative regulation of inflammatory response and in complement activation through the lectin pathway.

**Table 5:** List of the host proteins which were shown to have significantly increased or decreased expression (≥ 2- or 0.5-fold) in rabbits with convalescent *E*. *cuniculi* disease. Are shown in this table the 115-tagged proteins that significantly changed only in the 45 rabbits with convalescent condition consistent with past *E*. *cuniculi* infection.

Protein identification	Fold change	Known function	Chromosome	Gene name (GenBank^®^)	Access. number (UniProt^®^)	Subcellular location	Theoretical MW (kDa)	Calc. pI	Protein score (Sequest^®^)	No. unique peptide(s)	No. aa	% Seq. coverage
**SET domain bifurcated 2**	0.2	**transport and cell organization and biogenesis** (DNA binding, protein binding, metal ion binding); **metabolic process** (catalytic activity)	IX	***SETDB2***	G1TX00	Nucleus (chromosome)	81.8	8.0	5.1	1	719	3.2
**DEAH (Asp-Glu-Ala-His) box polypeptide 29**	0.3	**metabolic process** (catalytic activity); **transport** (RNA binding; nucleotide binding)	XI	***DHX29***	G1U383	Mitochondrion	105.6	8.2	8.3	2	1.366	3.4
**Keratin 1**	0.5	**defense response; metabolic process; regulation of biological process; response to stimulus** (structural molecule activity)	**IV**	*KRT1*	G1U9I8	Nucleus; membrane; blood microparticle; extracellular exosome, extracellular matrix; keratin filament	64.2	8.5	49.8	1	611	14.8
**Uncharacterized protein**	2.3	**Transport**		**N/A**	G1U0V3	Membrane	66.5	6.6	16.9	1	595	4.4
**Ig gamma chain C-region**	2.2	**Defense and transport** (protein binding)		**N/A**	P01870		35.4	8.3	37.6	2	323	24.5
**Immunoglobulin heavy-chain variable region**	3.7	**Defense and transport** (protein binding)		N/A	Q6B726		10.3	8.3	8.2	2	97	39.2
**Nudix hydrolase 11**	4.3	**metabolic process** (catalytic activity)		***NUDT11***	G1TGZ5		19.1	6.4	7.5	1	170	3.5

Abbreviations: aa, Amino acids; Access., Accession; calc., Calculated;

*, homologous protein; kDa, Kilo dalton; MW, Molecular weight; N/A, Not available; No, Number; pI, Isoelectric point; Seq., Sequence.

## Discussion

*Encephalitozoon cuniculi* is a microsporidian species common in rabbits and responsible for rare but serious infections in immunocompromised humans. It represents a major health issue for rabbits in which seroprevalence rates have been estimated by 37–68% of the pet population [24]. Although it has been largely studied for pathophysiology and immunology in experimental rodent models [9,10] and, to a limited point, in some experimentally-infected rabbits [8,25], additional insights are needed in naturally-infected hosts [26]. In the current study, we applied new mass spectrometry (MS) tools to comprehensively explore proteomic changes during *E*. *cuniculi* infection in naturally-infected rabbits. First, an *E*. *cuniculi* protein extract was used to select the Western blot (WB) bands that are targeted by the humoral immune response in infected rabbits. Thereafter, MS profiling identified the proteins in regions of the Coomassie-blue SDS-PAGE gel that corresponded to the bands detected in WB using rabbit sera. Although SDS-PAGE can display less resolution that two-dimensional gel (2D-gel) electrophoresis [19,27], the bands in this study allowed for clear selection for analysis. As the humoral response is theoretically only indicative of contact—current, recent or past—with the pathogen, including IgM titers which can persist over several months [6,28], it appeared relevant to focus not only on the description of antigenic microsporidian proteins but to comprehensively study all the changes in host responses in the pathophysiology of *E*. *cuniculi* infections in rabbits [6,8,29]. To address this, we conducted a direct differential comparison of proteomes *via* the iTRAQ^®^ protocol. The highly-sensitive iTRAQ^®^ reagents are particularly suited to this task as they can be used to simultaneously analyze samples from both active infection, past infection, and control conditions [15]. Unlike other proteomic methods as 2D-gels [19,30], this original tagging approach allows both identification and quantitation of all the over- or underrepresented proteins in the samples of interest, and not only the proteins that contain cysteine residues that are, for instance, the only ones amenable for labelling with alternative technology like the cleavable ICAT^®^ (Isotope-Coded Affinity Tag) reagents (Sigma-Aldrich, Saint-Louis MO, USA) [13,15]. Thus, there was theoretically no loss of information on post-translational modifications, like glycosylation or phosphorylation, or on small non-tryptic peptides [31]. The Orbitrap MS instrument afforded high resolving power (140,000 full width at half maximum, FWHM defined at *m*/*z* 200) and elevated resolution (Δ_*m*/*z*_ = 0.001) with a 50–4,000 *m*/*z* range [12]. All these technical features allowed for a distinct fractionating step, so that MS results were more reliable and accurate in comparison with standard Q-TOF devices with resolving power that usually spans from 22,500 to 60,000 FWHM, and resolution no lower than Δ_*m*/*z*_ = 0.02.

According to the WB patterns observed in seropositive rabbits (as well it was in infected dogs and cats, data not shown), eight bands were clearly highly and specifically antigenic. On Coomassie-blue SDS-PAGE gel, these regions corresponded to seven distinct proteins, plus one fragmented one. Notably, two were identified as proteins of *E*. *cuniculi* polar tube, polar tube protein 2 and polar tube protein *3* which have already been described previously [32,33]. Microsporidia, including *E*. *cuniculi*, invade their host cells through this unique cellular apparatus which has a great role in the pathophysiology [11], because it is able to discharge the microsporidian sporoplasm into the cell [34]. In addition, the involvement of spore wall and anchoring disk complex protein EnP1 in the humoral response of the host has not been described previously. This protein is expressed at the cell surface at the onset of microsporidian sporogony, and is associated with the chitin-rich layer of the cell wall in mature spores [35]. Its assumed role relies in cell adhesion and sporulation. Furthermore, we also described polysaccharide deacetylase domain-containing protein is targeted by the immune response perhaps because it acts during the sporogonial stage by protecting the microsporidian chitin layer from hydrolysis by mammalian chitinases. Its role in infection is currently still debatable [36]. Interestingly, we report that the other *E*. *cuniculi* proteins, revealed by our WB-MS approach, have putative functions that show they are rather involved in various metabolic processes and cell organization or catalytic activity including stress response and nucleotide binding. To date, this category of proteins has been far less described in immune response against *E*. *cuniculi* than structural proteins. For example, this is the first report that an endoplasmic reticulum-associated protein, like heat shock related 70kDa protein which is located in internal structures rather than at the spore surface, is reported in such a context in microsporidian species [37]. Similarly, heat shock protein 70 has already been shown to be regulated by stress response in some other invertebrates [38]. In addition, immune responses to zinc finger protein and translation elongation factor 1 alpha, have not been described before in *E*. *cuniculi* infections. Their reported function is so far only putative. It will be important to prospectively assess their antigenic potential in larger studies of infected rabbits since it appears that the detection of specific antibodies against these proteins, like anti-translation elongation factor 1 alpha, can distinguish between active and convalescent infection.

Pattern comparison for host protein expression by iTRAQ^®^ protocol was carried out in relation to a group of control rabbits with miscellaneous underlying inflammatory disease, including bacterial infection, otitis, urinary tract infection, meningoencephalitis, respiratory infection, which are conditions with similar clinical signs to microsporidiosis [6,39]. In this group, many proteins obviously appeared overexpressed *vs*. clinically normal rabbits, but we did not describe this in detail given our study mainly focused on rabbits with encephalitozoonosis. In the latter group, nine proteins were shown to be significantly increased. In addition to two proteins of the hemoglobin complex, as well as some others including immunoglobulin gamma-chain C-region and cystatin fetuin B-type, five had putative catalytic activity: alpha-1-antiproteinase precursor, alpha-1-antiproteinase S-1, haptoglobin, serum paraoxonase/arylesterase and ceruloplasmin. The former two both belong to the serine-protease inhibitor (serpin) superfamily, also referred to as serum trypsin inhibitors [40]. Such enzymes are usually overexpressed in extracellular space to protect host tissues from aggressive enzymes that are massively released during acute inflammation. In a previous study, incubation of *E*. *cuniculi* spores with trypsin was shown to have impact on their virulence [41], so it may be proposed that the increasing levels of host enzymes of the serpin family, like alpha-1-antiproteinase precursor and alpha-1-antiproteinase, may play a role in the *in vivo* regulation of the inflammatory response during encephalitozoonosis in rabbits. Paraoxonase is a high-density lipoprotein (HDL) associated with serum enzymes [42]. It can inactivate oxidized phospholipids carried by HDL and low-density lipoprotein [43]. Interestingly, resveratrol, a plant-derived phenolic compound, was previously shown to express an inhibitory action on *E*. *cuniculi*, and the paraoxonase was then thought to be involved in its possible pathway of action [44]. Haptoglobin and ceruloplasmin are transporter proteins that have also anti-inflammatory and anti-pathogen properties [45]. One of the main functions of haptoglobin is the clearance of free hemoglobin; thus, it may prevent the use of hemoglobin-associated iron which is probably crucial for *E*. *cuniculi* growth [46], as its sequestration was shown to contribute to macrophage-mediated control of the pathogen [47]. Furthermore, haptoglobin has immunomodulatory effects, in part due to its binding to CD11b-complement, and as it shifts the balance of T-cell cytokines in favor of TH_1_ immune response [48], which is essential for controlling *E*. *cuniculi* infection [6]. In our present study, we also noted the 2.6-fold increase of cystatin fetuin B-type protein. Cystatin fetuin proteins are enzyme regulators which were described to play an anti-inflammatory role during infection, although the A-type, also termed α-2-HS-glycoprotein for the human homologue, was the only one reported in similar context to confer a long-lasting protection against lethal sepsis in mice [49]. As suggested by other studies completed *in vitro* on *Aspergillus fumigatus*, cystatin fetuin A may also promote fungal growth [50]. However, our results provided new insights of cystatin fetuin involvement during inflammatory processes, as only little is known about the B-type protein during infection. Its possible role has so far been reported only in liver metabolic disease and myocardial infarction [51,52]. It may be valuable to prospectively assess the potential of one or several of all these proteins in combination as diagnostic biomarkers in a large validation cohort. In contrast, serum amyloid A (SAA) and C-reactive protein (CRP) were not reported in our work, while they were actually elevated in diseased rabbits with presumptive *E*. *cuniculi* active infection [53], Cray’s unpublished data]. This finding is due to the very stringent criteria of the bioinformatics parameters: for instance, false discovery rate was set at 1%, so that MS analysis highlighted only the proteins that were distinctly under- or overrepresented, and ignored those with smaller changes, like SAA and CRP.

Our iTRAQ^®^ approach for comparing the serum proteome of patients with different clinical signs of *E*. *cuniculi* infection [16] highlighted significant overabundance of some particular proteins during neurological involvement of encephalitozoonosis but not during the renal disease. Three of them were thought to be involved in the adaptive immunity through the classical pathway of the complement cascade, and four were enzyme regulators. Increased complement C4A, C3 alpha-chain and immunoglobulin heavy-constant mu in rabbits with well-developed disease was not surprising, since the role of antibody and complement in controlling *E*. *cuniculi* infection has been acknowledged previously [54]. Likewise, kininogen 1, alpha-2-macroglobulin, apolipoprotein A-I, and alpha-1-antiproteinase E have also been sporadically reported to be increased in infectious diseases [55], but their concomitant association which appeared specific during neurological clinical signalmen of *E*. *cuniculi* active infection is novel in such a context. Noteworthy, our findings about apolipoprotein A-I increase, a protein with well-known antioxidant and anti-inflammatory properties [56], were consistent with those of our prior article about aspergillosis in a rat model [19]. However, they appear contradictory with results from rabbits and mice models of aspergillosis with a significant decline [30,57]. These surprising discrepancies may reflect differences in the methods or animal models used, or the pathogens, although both are related to Fungi. The detection of increased kininogen 1 in the current study may be the result of a host defense mechanism of generating pro-inflammatory bradykinin-related peptides at the sites of infection, since some virulence factors, like secreted aspartic proteases of pathogens, were shown in an experimental model of candidiasis to induce host kininogen proteolysis. The released peptides thereafter recruit defense cells [58], and activate many types of cells to release further pro-inflammatory mediators [59]. Altogether, our data support the concept that protein patterns are differentially expressed in blood depending on the clinical course of infection, and may provide a new approach to understand host-pathogen interaction, detection, and prognostic stratification.

Proteomic studies have potentially-important diagnostic and pathophysiological implications for all infectious diseases. As a model, our original MS approach provided important new insights in the inflammatory and humoral immune response of rabbits with *E*. *cuniculi* infections. By displaying significant qualitative and quantitative changes in the blood proteome, it has illustrated protein functions and inflammation processes in such a context. It may also suggest new biomarkers which are essential for correct diagnosis and for monitoring therapeutic efficacy, and as well as targets for therapeutic intervention. Additional studies in experimental models are needed to confirm these results and expand on the construction of functional protein networks in health and disease.
